# Supplementary material for: Effect of Cooking with Superheated (SHS) vs. Standard Steam Oven on the Fatty Acids Profile of Different Kinds of Meat and Fish
Source: Foods. 2023 Feb 7;12(4):718. doi: 10.3390/foods12040718 (PMC9956209; doi:10.3390/foods12040718)
Supplement: Supplementary file 1 [file foods-12-00718-s001.zip › foods-2149440-SI.pdf]

Table S1. Fatty acid composition of different matrices (g/100 g of total fatty acids) *(first part)*

	BOV		BUR		SAL		POR		SE	Significance		
	SHS	SO	SHS	SO	SHS	SO	SHS	SO		M	C	M × C
C10	0.09	0.09	0.02	0.03	0.00	0.00	0.09	0.15	0.01	***	ns	ns
C12	0.10	0.09	0.07	0.07	0.05	0.03	0.07	0.11	0.00	***	ns	ns
C14iso	0.06	0.05	0.06	0.06	0.01	0.01	0.00	0.00	0.06	***	ns	ns
C14	2.51	2.50	2.41	2.46	1.87	1.83	1.12	1.40	0.01	***	ns	ns
C14-1t9	0.18	0.17	0.02	0.03	0.04	0.04	0.02	0.03	0.03	***	ns	ns
C15ante	0.32	0.27	0.23	0.24	0.00	0.00	0.00	0.00	0.02	***	ns	ns
C14-1c9	0.22	0.28	0.65	0.68	0.01	0.01	0.02	0.02	0.05	***	ns	ns
C15	0.42	0.38	0.50	0.50	0.14	0.14	0.03	0.04	0.01	***	ns	ns
C16iso	0.25	0.22	0.23	0.24	0.02	0.02	0.00	0.00	0.01	***	ns	ns
C16	23.17	23.67	24.27	24.48	8.63	8.45	23.08	24.25	0.32	***	ns	ns
C16-1c7	0.62	0.56	0.00	0.02	0.02	0.19	0.20	0.18	0.01	***	**	ns
C16-1c9	3.31	3.22	3.59	3.64	2.09	2.07	4.07	3.71	0.13	***	ns	ns
C17	0.95	0.90	0.94	0.96	0.26	0.26	0.21	0.25	0.02	***	ns	ns
C18iso	0.36	0.35	0.04	0.04	0.02	0.13	0.00	0.00	0.04	***	ns	ns
C17-1c9	0.39	0.35	0.58	0.66	0.16	0.11	0.24	0.23	0.06	***	ns	ns
C18	14.31	14.25	14.64	14.83	2.36	2.34	10.16	11.12	0.34	***	ns	ns
C18-1t6/8	0.11	0.09	0.21	0.22	0.00	0.00	0.10	0.07	0.01	***	ns	ns
C18-1t9	0.19	0.17	0.25	0.26	0.03	0.03	0.10	0.11	0.01	***	***	***
C18-1t10	0.16	0.14	0.46	0.49	0.00	0.00	0.00[b]	0.17[a]	0.01	***	***	ns
C18-1t11	0.89[a]	0.76[b]	1.65	1.69	0.00	0.00	0.00	0.03	0.03	***	ns	***
C18-1t12	0.19	0.18	0.33	0.35	0.00	0.00	0.00	0.00	0.01	***	ns	ns
C18-1c9	42.41	41.87	39.02	38.26	40.79	40.69	46.18	43.62	0.50	***	*	ns
C18-1c11	1.82	1.72	1.58	1.54	3.22	3.24	5.21	4.47	0.09	***	**	ns
C18-1c12	0.18	0.17	0.23	0.22	0.05	0.05	0.17	0.15	0.01	***	ns	ns
C18-2t11c15	0.07	0.05	0.13	0.16	0.00	0.00	0.00	0.03	0.01	***	ns	ns
C18-2cc	3.53	3.87	2.44	2.45	15.37	15.27	5.48	6.25	0.24	***	ns	ns
C20	0.09	0.08	0.14	0.14	0.28	0.27	0.15	0.18	0.01	***	ns	ns
C20-1c8	0.20	0.19	0.10	0.11	0.13	0.14	0.00	0.00	0.01	***	ns	ns
C18-3n3	0.64	0.62	0.54	0.56	5.74	5.79	0.25[b]	0.47[a]	0.03	***	ns	ns
CLA9-11ct	0.33	0.30	0.58	0.59	0.00	0.00	0.04	0.03	0.01	***	ns	ns
C20-2n6	0.06	0.06	0.03	0.03	1.01	1.02	0.25	0.23	0.01	***	ns	ns
C20-3n6	0.19	0.27	0.10	0.10	0.18	0.16	0.11	0.10	0.10	***	ns	ns
C20-3n3	0.00	0.00	0.02	0.02	0.37	0.44	0.08	0.09	0.01	***	*	*
C20-4n6	0.47	0.63	0.31	0.32	0.37	0.19	0.68	0.66	0.07	**	ns	ns

Table S1. Fatty acid composition of different matrices (g/100 g of total fatty acids) *(second part)*

	BOV		BUR		SAL		POR		SE	Significance		
	SHS	SO	SHS	SO	SHS	SO	SHS	SO		M	C	M × C
C20-4n6	0.47	0.63	0.31	0.32	0.37	0.19	0.68	0.66	0.07	**	ns	ns
C23	0.03	0.04	0.02	0.03	0.05	0.05	0.00	0.00	0.00	***	ns	ns
C20-5n3	0.02	0.05	0.05	0.05	2.63	2.69	0.03	0.04	0.02	***	**	ns
C22-4n6	0.07	0.08	0.06	0.06	0.03	0.00	0.15	0.12	0.02	***	ns	ns
C22-5n6	0.00	0.00	0.01	0.00	0.38	0.40	0.04	0.02	0.01	***	ns	ns
C22-5n3	0.23	0.30	0.16	0.17	1.20	1.21	0.12	0.14	0.02	***	ns	ns
C22-6n3	0.03	0.03	0.01	0.02	5.22	5.34	0.02	0.02	0.01	***	**	ns

BOV, bovine steak; BUR, hamburger; SAL, salmon; POR, pork steak; SE, standard error

Different letters within matrix correspond to different values for P < 0.05

Table S2. Classes of fatty acid composition of different matrices (g/100g of total fatty acids)

	BOV		BUR		SAL		POR		SE	Significance		
	SHS	SO	SHS	SO	SHS	SO	SHS	SO		M	C	M × C
SFA	42.66	42.89	44.84	45.15	14.01	13.82	35.06	37.59	0.50	***	ns	ns
MUFA	51.06	49.98	50.56	50.15	50.16	49.88	57.40	53.94	0.61	***	*	ns
PUFA	5.67	6.28	4.63	4.75	33.94	33.82	7.47	8.46	0.35	***	ns	ns
PUFA n6	4.33	4.93	3.00	3.01	17.58	17.26	6.79	7.49	0.31	***	ns	ns
PUFA n3	1.00	1.05	0.98	1.05	16.24	16.46	0.58	0.84	0.08	***	*	ns
$TFA_{18:1}$	1.54^{a}	1.35^{b}	3.37	3.47	0.02	0.03	0.20	0.38	0.05	***	ns	*
TFA	2.12^{a}	1.86^{b}	3.64	3.78	0.07	0.0.7	0.27	0.48	0.06	***	ns	*
BCFA	0.99	0.90	1.59	1.59	0.10	0.17	0.00	0.00	0.06	***	ns	ns
Total FA	78.73	71.89	80.89	77.92	85.03	86.12	70.72	67.84	1.71	***	*	ns

BOV, bovine steak; BUR, hamburger; SAL, salmon; POR, pork steak; SE, standard error

SFA, saturated fatty acids; PUFA, polyunsaturated fatty acids, MUFA, monounsaturated fatty acids; n-6, PUFA omega 6; PUFA omega 3; TFA, trans fatty acids; BCFA, branched chain fatty acid

Different letters within matrix correspond to different values for P < 0.05

Table S3. Fatty acid composition of different matrices (mg/100 g of meat) *(first part)*

	BOV		BUR		SAL		POR		SE	Significance		
	SHS	SO	SHS	SO	SHS	SO	SHS	SO		M	C	M × C
C10	3.58	3.30	1.59	2.32	0.00	0.00	5.54	8.49	0.70	***	ns	ns
C12	3.90	3.46	4.68	4.44	5.29	4.17	4.21	6.48	0.56	***	ns	ns
C14iso	2.30	1.90	3.68	4.02	1.02	0.56	0.00	0.00	0.19	***	ns	ns
C14	100.82	98.11	155.78	159.97	239.52	212.83	63.87	89.19	11.63	***	ns	ns
C14-1t9	6.92	6.27	1.60	1.21	5.71	5.18	1.52	1.50	0.36	***	ns	ns
C15ante	12.85	9.12	14.74	15.55	0.00	0.00	0.00	0.00	1.36	***	ns	ns
C14-1c9	11.52	13.75	42.17	43.84	1.66	1.93	1.18	1.39	2.62	***	ns	ns
C15	15.93	14.18	32.13	32.59	18.50	16.32	1.57	2.41	1.24	***	ns	ns
C16iso	9.18	8.30	14.92	15.58	2.59	2.27	0.00	0.00	0.62	***	ns	ns
C16	931.39	923.46	1586.92	1588.76	1100.35	979.08	1321.03	1567.45	139.34	***	ns	ns
C16-1c7	23.73	21.10	0.00	1.62	25.11	20.07	11.58	11.15	1.83	***	ns	ns
C16-1c9	141.70	126.27	232.72	236.01	266.63	239.84	233.69	230.08	20.79	***	ns	ns
C17	35.96	34.24	60.75	62.56	29.94	29.84	12.15	15.94	2.88	***	ns	ns
C18iso	11.05	10.97	2.28	2.15	2.48[b]	15.15[a]	0.00	0.00	0.94	***	***	***
C17-1c9	20.24	16.93	37.42	37.98	20.73	12.25	13.82	14.87	4.03	***	ns	ns
C18	540.75	538.62	948.24	963.48	301.51	270.45	579.19	726.25	67.23	***	ns	ns
C18-1t6/8	3.94	3.14	13.56	14.31	0.00	0.00	5.85	4.50	0.57	***	ns	ns
C18-1t9	7.49	6.76	16.16	16.87	2.85	3.11	5.52	7.31	0.85	***	ns	ns
C18-1t10	6.20	5.88	29.69	31.51	0.00	0.00	0.00[b]	10.26[a]	1.17	***	ns	**
C18-1t11	32.60	28.05	107.04	109.91	0.22	0.00	0.00	1.52	3.40	***	ns	ns
C18-1t12	7.21	6.84	21.16	22.54	0.00	0.00	0.00	0.00	0.71	***	ns	ns
C18-1c9	1686.59	1592.51	2533.42	2480.05	5217.10	4703.37	2668.67	2861.06	275.39	***	ns	ns
C18-1c11	77.03	66.76	103.03	99.64	412.11	374.89	300.81	282.63	22.96	***	ns	ns
C18-1c12	6.51	5.94	14.75	14.58	6.83	5.67	10.18	9.32	0.83	***	ns	ns
C18-2t11c15	2.24	1.75	8.50	10.38	0.00	0.00	0.00	1.93	0.70	***	ns	ns
C18-2cc	133.26	137.13	158.70	158.46	1966.87	1761.56	309.91	367.29	45.68	***	ns	ns
C20	4.14	3.77	8.82	9.49	36.40	31.70	8.86	12.65	1.59	***	ns	ns
C20-1c8	6.95	6.59	6.81	7.16	17.36	15.72	0.00	0.00	0.65	***	ns	ns
C18-3n3	23.94	22.53	35.01	36.17	735.10	667.88	14.41	28.75	15.79	***	ns	ns
CLA9-11ct	13.98	11.49	37.91	38.44	0.00	0.00	3.00	2.93	1.44	***	ns	ns
C20-2n6	2.44	2.26	2.19	2.16	130.17	117.02	14.64	15.06	2.16	***	ns	ns
C20-3n6	7.85	8.94	6.40	6.78	23.08	18.80	6.30	5.50	5.50	***	ns	ns
C20-3n3	0.00	0.00	1.03	1.15	48.49	50.70	4.87	6.25	1.03	***	ns	ns

Table S3. Fatty acid composition of different matrices (mg/100 g of meat) *(second part)*

	BOV		BUR		SAL		POR		SE	Significance		
	SHS	SO	SHS	SO	SHS	SO	SHS	SO		M	C	M × C
C20-4n6	19.33	22.80	20.39	21.10	43.47	21.63	36.42	33.87	3.93	**	ns	ns
C23	1.03	1.21	1.66	1.81	7.04	5.52	0.00	0.00	0.25	***	ns	ns
C20-5n3	1.06	1.84	3.29	3.51	334.31	310.98	1.96	2.06	6.54	***	ns	ns
C22-4n6	3.80	3.52	3.81	3.62	4.37	0.00	8.99	7.03	1.30	***	ns	ns
C22-5n6	0.02	0.09	0.84	0.15	47.82	46.38	2.34	1.22	0.80	***	ns	ns
C22-5n3	9.67	10.74	10.72	11.38	153.04	139.58	6.88	8.02	3.02	***	ns	ns
C22-6n3	0.92	0.85	1.05	1.29	664.00	615.20	1.28	1.30	11.52	***	ns	ns

BOV, bovine steak; BUR, hamburger; SAL, salmon; POR, pork steak; SE, standard error

Different letters within matrix correspond to different values for P < 0.05

Table S4. - Classes of fatty acid composition of different matrices (mg/100g of meat)

	BOV		BUR		SAL		POR		SE	Significance		
	SHS	SO	SHS	SO	SHS	SO	SHS	SO		M	C	M × C
SFA	1673.23	1650.66	2905.82	2931.97	1782.55	1601.07	2004.70	2433.41	222.51	***	ns	ns
MUFA	2045.41	1910.78	3281.43	3245.54	6415.55	5767.14	3317.30	3513.15	336.69	***	ns	ns
PUFA	219.20	224.88	300.48	307.74	4332.22	3904.36	421.65	494.23	90.39	***	ns	ns
PUFA n6	167.28	175.54	194.96	195.27	2245.69	1991.27	382.98	435.98	50.74	***	ns	ns
PUFA n3	37.84	37.71	63.52	68.05	2072.25	1900.97	33.01	51.00	41.30	***	ns	ns
$TFA_{18:1}$	57.43	50.67	217.66	225.75	3.07	3.11	11.37	24.17	6.82	***	ns	ns
TFA	80.57	70.18	235.63	245.19	8.78	8.30	15.89	29.97	7.79	***	ns	ns
BCFA	35.51	30.29	102.62	104.10	13.28	19.32	0.00	0.15	3.77	***	ns	ns
Total FA	3938.09	3786.43	6489.36	6487.85	12615.67	11365.94	5743.65	6440.80	609.97	***	ns	ns

BOV, bovine steak; BUR, hamburger; SAL, salmon; POR, pork steak; SE, standard error

SFA, saturated fatty acids; PUFA, polyunsaturated fatty acids, MUFA, monounsaturated fatty acids; n-6, PUFA omega 6; PUFA omega 3; TFA, trans fatty acids; BCFA, branched chain fatty acid

Different letters within matrix correspond to different values for P < 0.05
